# Intra-Varietal Variability for Abiotic Stress Tolerance Traits in the Grapevine Variety Arinto

**DOI:** 10.3390/plants14162480

**Published:** 2025-08-10

**Authors:** Luisa C. Carvalho, Teresa Pinto, Joaquim Miguel Costa, Antero Martins, Sara Amâncio, Elsa Gonçalves

**Affiliations:** 1 LEAF-Linking Landscape, Environment, Agriculture and Food, Associate Laboratory TERRA, Instituto Superior de Agronomia, Universidade de Lisboa, Tapada da Ajuda, 1349-017 Lisboa, Portugalelsagoncalves@isa.ulisboa.pt (E.G.); 2 Associação Portuguesa para a Diversidade da Videira-PORVID, Tapada da Ajuda, 1349-017 Lisboa, Portugal

**Keywords:** empirical best linear unbiased predictors (EBLUP of genotypic effects), polyclonal selection, quality of the must, SLT (surface leaf temperature), yield

## Abstract

The valorization of genetic intravarietal variability through the identification of the most suitable genotypes for yield and must quality is an adequate strategy for grapevine selection. Currently, climate change affects vine yield and wine quality in numerous ways, but little information is available on intravarietal variability regarding responses to abiotic stresses. In the current work, the intravarietal genetic variability of the Portuguese white variety Arinto was studied for yield, must quality, and for tolerance to abiotic stress, through indirect, rapid, and nondestructive measurements in the field. An innovative approach in selection for abiotic stress tolerance is described. The surface leaf temperature (SLT) of clones under environmental conditions of drought and extreme heat was measured, as were the NDVI (Normalized Difference Vegetation Index); PRI (Photochemical Reflectance Index); and chlorophyll content through the SPAD index, yield, and the characteristics of the must (pH, acidity, and °Brix). The application of this methodology was carried out in an experimental population of 165 Arinto clones for three years. Linear mixed models were fitted to the data from evaluated traits, and the empirical best linear unbiased predictors (EBLUPs) of genotypic effects were obtained, as well as the coefficient of genotypic variation (CVG) and broad-sense heritability. The genotypes were ranked according to their level of tolerance to abiotic stress without loss of yield/quality. SLT enabled the identification of clones that regulate stomata opening during stress, thus correlating positively with yield. SLT appears, thus, to be the most robust and reliable indicator to assess tolerance to stress in large field trials for grapevine selection. The results enabled the selection of a group of ten clones with increased tolerance to stress, compared to the average of the variety which maintained the typical must quality of Arinto.

## 1. Introduction

The culture of grapevines for wine production is a major source of economical revenue in Mediterranean-area countries, Portugal among them. In fact, in 2022 Portugal was the tenth wine producer worldwide, with 6.8 million hL [1]. Furthermore, Portugal has a very high biodiversity within the *Vitis vinifera* species, with 265 officially described Portuguese varieties in 2020 [2], that are themselves represented by hundreds of genotypes (termed clones), that show different values of the most important economic traits (such as berry composition and yield).

Climate change in the Mediterranean area raises air temperature, intensifies water scarcity, and increases the frequency of extreme weather events (such as severe drought and heat waves), that are affecting viticulture [3]. Irrigation is a good strategy to overcome punctual shortcomings, but in the long run is not sustainable, as water availability dwindles. Therefore, a sustainable and effective strategy to maintain viticulture in those areas is to explore the intra-varietal variability that exists between the clones of each variety to choose the ones that are best adapted to withstand climate extremes.

A bottleneck of this approach is the need to individually phenotype hundreds of clones, which, with the appropriate statistical design of the experimental fields results in thousands of plants to measure. As these trials are so extensive, the use of aerial or satellite imaging would be ideal. However, currently, they do not have resolutions sufficient to assess differences between clones [4]. The measurement of canopy temperatures through infrared thermography has some potential for plant phenotyping in the field and assesses response to abiotic stress, mainly to drought [5,6] and yield [6]. These parameters are closely related to the phenomena of plant evaporative cooling, because when stomata are closed, leaf temperature increases, and when they are open, the leaf transpires and cools down, at the expense of losing water. This, in turn, reflects plant water availability, because to enable evaporative cooling, the plant needs to have some water available, but it also reflects the ability of the plant to control water loss even at low water availability. Also, when stomata close, CO_2_ intake for photosynthesis becomes minimal and carbohydrate production is affected.

Thermal cameras recording electromagnetic radiation in the infrared region, where most of the heat is located in the electromagnetic spectrum, can be used to assess leaf temperature [7]. These images have proven to be suitable for different plant species and for different conditions [6], especially when studying the effect of a stress treatment. However, when the objective is to assess the response of many genotypes to the same environmental conditions, the sensitivity of the method is not enough to achieve an accurate characterization. Therefore, another leaf temperature related parameter, surface leaf temperature (SLT), has been developed, which showed good results in genotypes of the variety Aragonez (syn. Tempranillo) [8].

Other possibly interesting phenotyping tools available are in planta quantifications of reflectance indices and non-destructive chlorophyll content measurements. The latter, the SPAD index, gives a measure of chlorophyll concentration in leaves [9], and so does the reflectance index, the NDVI (Normalized Difference Vegetation Index). Both are good indicators of vigor and, reflected by a decrease in their values, of stress. The NDVI has been used for selection of wheat genotypes based on yield, under various environmental conditions [10,11,12,13,14]. The reflectance index, the PRI (Photochemical Reflectance Index), measures anthocyanins; more specifically, the functioning of the xanthophyll cycle. It is, therefore, an indicator of the plant’s response to environmental conditions [15], specifically its ability to scavenge reactive oxygen species (ROS) accumulated under stress, and ultimately, of its tolerance to withstand that stress. Conversely, SLT values reflect the ability of stomata to remain open, even under adverse environmental conditions of water scarcity and high temperatures. This ability correlates directly with yield, as it enables a steady flux of CO_2_ for photosynthesis.

The methods of selection of grapevine clones involve a comparative analysis of hundreds of genotypes, that are present in several biological replicates in an experimental field [16,17,18]. When the yield and quality of the must are quantified, eventual changes due to the time of day of sampling are not significant and will not alter the results. However, when quantifying the plant’s response to an environmental condition, such as water and heat stress, that response is itself dependent on the environmental condition, which is not stable for a long period during the day [8]. Therefore, one of the most challenging aspects of selection to characterize abiotic stress response is plant phenotyping work. This makes the measurement of some important but time-consuming physiological parameters impractical and sometimes even unfeasible. It is thus paramount to develop expeditious and accurate measurements for field phenotyping.

Arinto is a white grapevine variety that has a long tradition in Portugal, particularly in the wine-producing regions of Lisboa (Bucelas), Bairrada, Lafões, and Vinhos Verdes. The Arinto grape is a late-budding and ripening variety, exhibiting high acidity, small berries with a favorable skin-to-flesh ratio, and a satisfactory yield [19]. There is a broad consensus within the Portuguese vitivinicultural sector is that the Arinto variety has notable oenological value and the potential to enhance the quality of wines from numerous regions when employed in blends with other varieties. The acknowledgement of its significant oenological potential, coupled with the current tendency towards fresh, aromatic, and high-quality wines, has resulted in its expansion across all wine-producing regions. In 2022, it was the 3rd most cultivated white variety in Portugal, representing 4% of the total production, while in Vinhos Verdes Arinto ranked 2nd, at 15%, and in Alentejo it was the 2nd white variety, with 3.9% of the total share [20]. However, the widely praised acidity characteristic of this variety is likely threatened by climate change [21,22].

In this work, a collection of Arinto clones established in an experimental field for selection of this variety was evaluated. This field is located in the Setúbal District, in the Experimental Centre for the Conservation of Grapevine Intravarietal Diversity of PORVID (Portuguese Association for Grapevine Diversity) and comprises 165 genotypes of this variety. These genotypes were used for stress tolerance studies during the seasons of 2019, 2020, and 2021. Monitoring was based on the identification of genotypes with lower SLTs, better values of the Normalized Difference Vegetative Index (NDVI), the Photochemical Reflectance Index (PRI), and chlorophyll content measured through the SPAD index. These were complemented with analyses of yield and berry characteristics. SLT and the indices were used as non-invasive and expedite indicators of abiotic stress tolerance and the accuracy and reproducibility of the results is discussed, as well as the efficacy of the method used to identify the clones that are tolerant to stress, regardless of environmental conditions and, among those, the ones that give rise to musts with good quality traits for wine production.

## 2. Results

### 2.1. Genetic Variance Components Estimates and Quantification of Intra-Varietal Variability

The estimate of the genetic variance component enabled the quantification of the genetic variability within the variety for all the traits in the three seasons of measure of the abiotic stress indicators. The results of the analysis (Table 1) showed that there was significant genetic variability within the variety for all traits and years (*p* < 0.05), although at different levels depending on the trait. According to the values of the genotypic coefficient of variation obtained for the field trial, higher intra-varietal variability was found for yield, acidity, berry weight, PRI, and SPAD, and the lowest for NVDI.

In the analysis of the percentage of the total design effect variance of the data attributable to each design effect, the importance of the effects of the experimental design depended on the trait and the year evaluated. The effects were generally more important for the resolvable replicate effects and the columns within resolvable replicate effects (Table 1). In particular, the greater percentage of variability in the design effects for the abiotic stress indicators is attributed to resolvable replicate effects (which also include the effect of the day) and the columns within replicate effects (which also include the time of day). The percentage of variability explained by each of these factors was dependent on the year; in conditions that were more stressful (e.g., 2019), the importance of the effects of the resolvable replicates increased, particularly for SLT. Regarding the other traits analyzed, in particular yield, berry weight, and soluble solids, the proportion of variability that was attributed to rows within replicate effects (largely associated with the control of spatial variability) exceeded that of the abiotic stress indicators. For the evaluation of berry pH, the effect of the columns within replicates was the most important to consider in the model for data analysis.

The proportion of phenotypic variance that can be explained by genotypic variance is also provided in Table 1, as indicated by the values of generalized broad-sense heritability (H^2^). NDVI and PRI had the lowest values of H^2^ (approximately 0.2 and 0.3, respectively, in the two seasons with lower levels of stress, 2020 and 2021), while the values of H^2^ obtained for SPAD and SLT were moderate to high (always >0.45), and equivalent to those of yield and berry traits.

The difference between the EBLUP of the genotypic effect and the PGVs of the clones with the highest and lowest values for all the traits in the three seasons are shown in Table 2. The NDVI had lower values in 2019, but with a larger difference between the lowest and highest values than in 2020, where the range of variation between clones was only 0.013, compared with 0.041 in 2019. PRI also had the highest values in 2021 and the largest difference between the lowest and highest values. In 2019 and 2020, the results were very similar, with a minimal range of variation between clones. SPAD, on the other hand, showed a higher difference between maximum and minimum values in all the seasons, with genetic quantifiable differences of five to six SPAD units between the best and worst-performing clones. Considering SLT, there was a genetic quantifiable difference of 5 °C between the coolest and warmest of the 165 clones measured in 2019, while that difference decreased to around 3 °C in the two following seasons. Maximum and minimum PGVs for SLT also decreased from 2019 to 2021. Yield followed a different pattern, with minimum PGVs constant throughout the season and only the maximum values increasing from 2019 to 2021; consequently, the differences between minimum and maximum EBLUPs also increased. Berry traits, however, were more conserved along the three seasons measured, regarding the span of EBLUP and PGVs, except for total acidity, which had lower minimum and maximum PGV in 2020.

For the three seasons, correlations between PGV of all the traits are described in Supplemental Figure S1 The correlation between abiotic stress indicators and the yield and berry traits evaluated varied between weak and moderate, depending on the year. This was observed even for correlations between traits that were significantly different from zero.

The estimated genetic correlations between the three years for the different traits and abiotic stress indicators evaluated are shown in Table 3. The genetic correlations found were lower for SLT between the three years, indicating that, among the abiotic stress indicators evaluated, this was the one that showed a higher genotype × year (G × Y) interaction. For the other abiotic stress indicators, the multi-trait mixed model fitted to estimate these correlations revealed convergence problems, likely due to the low range of variation in the measurements associated with PRI, NDVI, and SPAD. For yield and berry traits, the genetic correlations between years were generally high, showing that the G × Y interactions for these traits were lower than the ones obtained for SLT.

### 2.2. Polyclonal Selection

**Figure 1:**
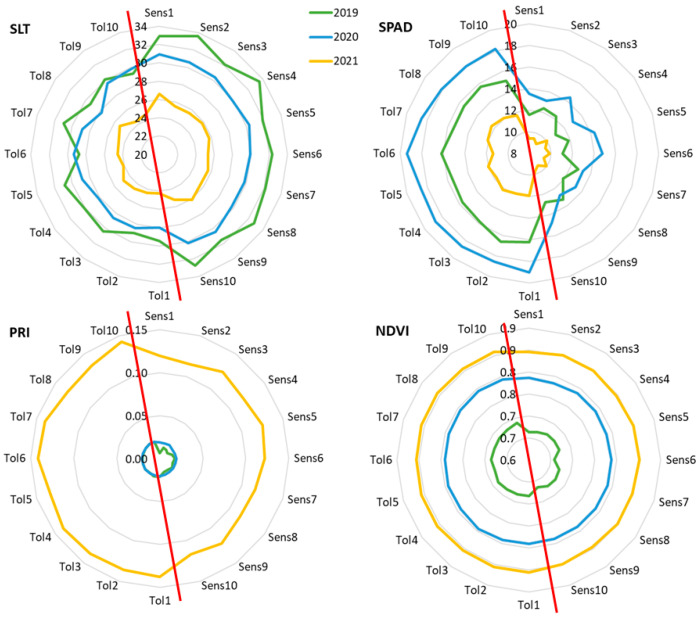
Graphical representation of the predicted genotypic values for the ten best and worst ranking clones after selection for each of the abiotic stress indicators NDVI, PRI, SLT, and SPAD. The red line separates tolerant and sensitive genotypes, according to the respective stress indicator. Identification of the clones is highlighted in Supplementary Table S2.

For the polyclonal selection, the 165 genotypes were ranked according to the Empirical best linear unbiased predictors (EBLUPs) of genotypic effects and the predicted genotypic values (PGVs) for SLT, SPAD, NDVI, and PRI. The values of these parameters for all the abiotic stress indicators in all the genotypes in 2019, 2020, and 2021 are provided in Supplementary Table S1 (EBLUPs) and Table S2 (PGVs and the rank for each trait). The ten top-ranking genotypes (termed Tolerant) and the ten worst-performing ones (named Sensitive) for each stress indicator were compared across the three seasons (Figure 1). There were two different patterns, the SLT and SPAD showing visible differences between the group of tolerant and sensitive clones in the three seasons, while NDVI and PRI had clearly different values between seasons and the differences between sensitive and tolerant clones were only visible in 2019 (NDVI and PRI) and 2021 (only PRI).

To understand the performance of the material selected according to each abiotic stress indicator, a first exercise of selection was conducted. Here, the groups of ten topmost genotypes for each abiotic stress trait in each of the seasons were used for selection. The predicted genetic gains of those selected groups were calculated for the respective abiotic stress indicator and for yield and berry traits (Table 4). The values of the estimates of the genotypic variance components (${\hat{\sigma }}_{g}^{2}$), the coefficient of genotypic variation (CVG), and the broad-sense heritability (H^2^) obtained for the average of the years where H^2^ was higher than 0.40 are presented in Supplementary Table S3. Selection according to NDVI gave rise to significant but nevertheless small gains in the parameter itself in the three seasons, while yield increased significantly in 2021 and acidity decreased slightly in 2019. Conversely, selection using PRI or SPAD provided significant gains in the parameters themselves in all the seasons but no significant increases in yield or berry traits (with the exceptions of pH in 2019 for both and °Brix in 2020 for SPAD). Finally, selection according to SLT led to better performance of the parameter itself in the three seasons, significant increases in yield, pH and °Brix and a decrease in TA, in 2019.

**Figure 2:**
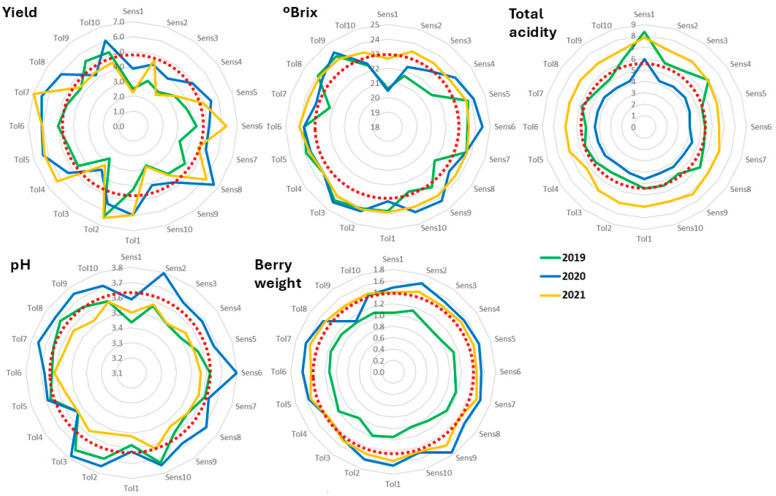
Graphical representation of the predicted genotypic values of yield and berry traits of the ten best- and worst-ranking clones after selection for SLT. The red dotted circle represents the global average of the PGV of the respective trait. Identification of the clones is highlighted in Supplementary Table S2.

The abiotic stress indicator that obtained a selected group of genotypes with simultaneous significant gains in the indicator and in yield and berry traits was SLT, especially in 2019. Therefore, the final polyclonal selection of a group of ten genotypes was conducted according to the EBLUPs of genotypic effects of SLT obtained from the analysis using the mean values from the three seasons. The predicted genetic gains for all traits and stress indicators were then calculated using data from the average of all the seasons monitored (Table 5). Compared to the mean behavior of the variety in the field trial, the SLT of the selected group decreased by 3% while SPAD increased by 3.2%; yield and °Brix also increased significantly, and the other traits and indicators did not change significantly (Table 5). This pattern becomes apparent in Figure 2, where the ten top-ranking genotypes (termed Tolerant) and the ten worst-performing ones (named Sensitive) for SLT were analyzed for yield and berry traits in the three seasons. Yield had a tendency to be higher than the global average in the tolerant clones while the sensitive ones had lower than average values. Conversely, berry traits did not seem to be affected by the level of tolerance of the clones, with patterns that were almost circular and, in general, coincided with the global average of the parameter.

## 3. Discussion

### 3.1. The Need for Abiotic Stress Tolerant Material

The high levels of intra-varietal variability within ancient grapevine varieties have been used in selection methods for decades [23]. So far, this selection has focused mostly on the yield and quality traits of the berries to increase or decrease the levels of sugars, acidity, and aroma compounds in musts [24,25]. Climate change threatens traditional viticulture in mediterranean climate areas, with increasing water scarcity and higher frequency of extreme events such as heat waves in the summer. Thus, the question arises as to whether this intra-varietal variability can also be used to obtain material that is tolerant to these abiotic challenges. The answer to this question is yes, as several studies have already shown [4,8,21,26]. However, it is still not clear what parameters to evaluate, nor how they affect yield and quality, although the parameter surface leaf temperature (SLT) seems a good candidate [8]. Therefore, in the current research, an evaluation of several indicators related to vigor and abiotic stress, which can be measured in an expedited way in experimental fields with thousands of plants, was performed, simultaneously with yield and berry characteristics.

An experimental field for the variety Arinto was used, set up with a specific layout that makes it possible to control for the effects of the environment and, simultaneously, to quantify the contribution of the genetic component through broad-sense heritability and the EBLUPs of genotypic effects, as previously reported by Tempranillo [8]. The findings of the present study demonstrated that a well-planned methodology can be effectively implemented to address this increasing need in the vitiviniculture sector.

### 3.2. The Importance of the Experimental Design

This study clearly demonstrated the critical role of experimental design in field trials. The effectiveness of the design and the significance of specific design factors varied depending on the year and the trait under investigation. This is justified by the effects of cultivation techniques, biotic and abiotic stresses, and other specific contexts associated with each year and trait. Similar results were obtained by Gonçalves et al. [27] for yield in several field trials of different grapevine varieties. The current work extends these findings by also investigating physiological and berry traits, demonstrating the usefulness of resolvable row–column designs in grapevine field trials for selection where a wide range of traits and years can be evaluated.

### 3.3. Abiotic Stress Tolerance Indicators

In the current study, the already-used parameter SLT [8], together with SPAD, PRI, and NDVI, was used. The quantifications of the abiotic stress indicators were undertaken during the seasons of 2019, 2020, and 2021; three years where meteorological conditions at the experimental site were similar regarding average and maximum air temperatures and winter rainfall (Figure S1), while water availability in the vineyard was lower in 2019 than in the two subsequent years.

Broad-sense heritability (H^2^) is a particularly useful concept in the field of quantitative genetic analysis, as it reflects the relationship between the real and the predicted genotypic effects. Consequently, it can be used as an indicator of the success of genetic selection. Broad-sense heritability was, on average, higher for SLT and SPAD; a very interesting result, given that the traits in question are subject to a high degree of extraneous variability. The values of H^2^ for these parameters were also affected by stress, being lower in 2021. In this season, it was harder to distinguish between genotypes, and consequently, the genetic variability found within the variety was lower. This was also demonstrated by the lower range of values obtained for the EBLUPs of the genotypic effects between the sensitive and tolerant genotypes in 2020 and 2021. Also, the EBLUPs were lower for NDVI than for the other parameters.

When comparing the results obtained for the abiotic stress indicators, there were two levels of genetic variability within the variety. Lower values were obtained for NDVI and PRI, while SLT and SPAD showed higher genetic variability in the three seasons, similar to the levels obtained for yield and berry traits. In the case of NDVI, the range of the values obtained for the clones was very small, with all the values close to each other; an indication that the parameter is not sensitive to changes within the variety. It is thus not good to identify differences within the variety. In fact, it is well known that chlorophyll-related indexes, such as NDVI, although often described as stress responsive, are indeed conserved, and in grapevine have been used for varietal discrimination [28,29,30].

For each trait, the changes in the quantification of intra-varietal variability between years result from a certain degree of genotype x year (GxY) interaction, exposed by the genetic correlations estimated between years (Table 3). High levels of correlation between different years are an indication that the clones evaluated are responding in the same way to the environmental changes and thus are stable across different environments regarding that characteristic. Among the abiotic stress indicators evaluated, SLT had the highest GxY interaction, an indication of a higher degree of distinct responses to the environment. Also, in the season with higher levels of stress, 2019, more genetic variability was identified due to a higher expression of the capacity to withstand stress, and thus, more ability to distinguish between clones. In fact, greater or lesser responses to GxY depend on how each genotype responds to stress. Usually, the higher the stress, the higher the intra-varietal genotypic variability estimated for that year.

### 3.4. Predicted Genetic Gains of Selection

The predicted genetic gains as a percentage of the mean of the population (Rs) were calculated for all the abiotic stress indicators in the three seasons, and for yield and berry traits when choosing the ten best-performing genotypes according to that indicator. The highest percentages of Rs were obtained in 2019 for all indicators; possibly a reflection of the highest levels of stress, particularly water stress, during that season. In fact, under conditions of stress, Rs values for the selection of the most tolerant group were *circa* twice the Rs obtained when selection was undertaken without stress. This was so for all the indicators, except SPAD, where the increase in Rs was of only 20%.

Regarding yield, the genotypes chosen through SLT in 2019 gave rise to the highest gains, followed by those selected through PRI. Conversely, NDVI and SPAD led to the highest gains in yield in 2021. This may not be related to a response to stress but to a positive correlation between yield and vegetative vigor, measured through the reflectance indices based on chlorophyll content [31,32,33]. What these parameters are showing, therefore, is a consequence of the lack of stress in 2021, while in 2019 and 2020, selection according to these indicators led to no significant changes in yield in the selected polyclonal group.

As for berry traits, none of the indicators chose polyclonal material with consistent significant changes in any of the traits in all the seasons, with a few exceptions, namely, °Brix, pH, and acidity for SLT, and °Brix and pH for NDVI, all in 2019. This indicates that, on average, the general performance of the variety will not be affected when using any of those indicators to select for stress tolerance, as was also reported in Tempranillo [8]. When exploring the genetic variability within the variety, its typical berry traits can be preserved when selecting genotypes that are tolerant to abiotic stress.

### 3.5. Polyclonal Selected Material

The high values of predicted genetic gains as percentages of the mean of the population (Rs) for yield when selecting according to SLT and the low levels of estimated genetic correlations between the three years suggest that this indicator had the highest G×Y interaction, and that, therefore, was showing a response to the different stress conditions of each season. Thus, SLT was the indicator that best identified tolerance to stress. This was especially evident in the season with the most stressful environmental conditions, 2019, when summer heat was combined with low water availability in the soil. Under these stressful conditions, tolerant and sensitive clones were better distinguished, leading to the detection of a higher level of genetic variability. The clones selected based on the most stress may partially lose their competitive advantage in non-stressful years. Selection should therefore include data from average and less stress-prone conditions, to obtain more balanced material that, on average, will be more tolerant. With this in mind, polyclonal selection of the ten best-performing clones was carried out according to the average of the three seasons based on SLT. Genetically, this material has a prediction of a 3% decrease in SLT with 95% confidence, on average, in relation to the mean of the variety. Nevertheless, in conditions of severe stress, the gain is expected to be higher, with a higher decrease in SLT expected to be observed, closer to the lower limit of the interval of prediction. Therefore, under conditions of stress, this gain can enable the cultivation of the polyclonal-selected material in areas of extreme weather conditions in which the average material of the variety cannot currently be grown. The mean behavior of the polyclonal-selected material for more tolerance also enabled a significant increase in yield with a mean predicted genetic gain of *circa* 7%, while the berry traits typical of the variety were not altered in the selected material. Arinto already has very good oenological characteristics, with high acidity; its use being recommended to face climate change, to counter the typical increase in sugar content and decrease in acidity already taking place [21,34]. With this selected material the variety becomes more tolerant and more productive while keeping its typical profile, leveraging its potential use in the future.

### 3.6. Why Was SLT the Best Stress Indicator?

The analysis of SLT is based on the assessment of stomatal regulation, which affects leaf temperature by controlling transpiration rates. Efficient stomatal conductance helps in cooling the leaf, termed evaporative cooling, thereby enhancing heat tolerance. Through this process, a plant can maintain its leaf temperature lower than the atmosphere’s when under heat stress. For this process to be effective, especially under conditions of water shortage, the plant must achieve good control of stomatal opening. In fact, plants that can keep lower leaf temperatures under those conditions are better adapted to such environments, and thus the quantification of leaf temperature can assess the combined effects of heat and drought [35,36]. Furthermore, the good control of stomatal opening, in turn, will be related to a better modulation of gas exchange and thus of CO_2_ uptake [7]. Therefore, when selecting for SLT, yield should also increase, as was in fact the case.

## 4. Materials and Methods

### 4.1. Description of the Field Trial and Experimental Design

The field trial was established in 2013 at the Experimental Centre of the Portuguese Association for Grapevine Diversity in Pegões (38°38′55.2″ N, 8°38′32.1″ W; altitude 69 m; district of Setúbal, southern Portugal). The climate of the region is Mediterranean with hot and dry summers and mild winters. The average annual rainfall is 550 mm, 400 of which usually fall during autumn and winter. The soil is mostly sandy with some clay-rich spots and is derived from podzols. This field trial contains a representative sample of the intra-varietal variability of the variety Arinto, composed of 165 accessions prospected in the different ancient growing regions of this variety in Portugal (Bairrada, Lafões, Lisboa, and Vinhos Verdes). All accessions were identified as the Arinto variety through ampelographic description. In addition, their varietal identity was confirmed using nine microsatellite markers (SSRs), in accordance with OIV recommendations for the genetic identification of grapevine varieties [37].

The field trial has been established in accordance with a resolvable row–column design (incomplete blocks in row and column directions within each resolvable replicate): 6 resolvable replicates, 11 rows, and 15 columns nested within each resolvable replicate, and 3 plants per experimental unit. This type of experimental design plays a key role in controlling for spatial variability, water regimes, management practices, and other sources of non-genetic variation in the field trial. All plants were grafted on the same clone of rootstock 1103P and were free from leafroll-associated virus type 3 (GLRV-3) and grapevine fanleaf virus (GFLV). The training system is a vertical shoot position, the pruning system is a bilateral Royat cordon system, and the planting density is 2.50 m × 1.20 m. Throughout all vegetative cycles, vineyard management followed cultural practices and phytosanitary treatments appropriate to the conditions of each year, ensuring proper vineyard maintenance.

### 4.2. Environmental Conditions and Abiotic Stress Evaluation

The evaluations were conducted in the seasons of 2019, 2020, and 2021. The field was not irrigated in 2019, and in the two following seasons, drip irrigation was applied. In 2019 Ψ_pd_ (pre-dawn leaf water potential) was measured (pressure chamber, Model 600, PMS Instruments Company, Albany, OR, USA) and the average value obtained was −0.7 MPa. In 2020 and 2021, irrigation was 3800 m^3^ ha^−1^ year^−1^, and was withheld two weeks prior to measurements, with average values of Ψ_pd_ of −0.3 MPa. The monthly average highest and lowest air temperatures were very similar in the three seasons (Supplemental Figure S2). There was very little rainfall in the winter–spring of 2019, but in 2020 there were very high levels of rainfall in spring, and in 2021, heavy rains in February were followed by very little rainfall (Supplemental Figure S2).

For the evaluation of all abiotic stress indicators, measurements were taken at peak heat hours on leaves exposed to the sun. The structure of the experimental design of the field trial was crucial in controlling for the environmental conditions, namely the effects of day and time of evaluation. Each resolvable replicate was evaluated on one day and in each day the plants of the experimental units of each column were measured in the shortest possible time. Therefore, each resolvable replicate also included the effect of day and each column within each resolvable replicate included the effect of time of day. In each experimental unit, three measurements were performed on three different leaves with one measurement for all the parameters except SLT, with ten technical replicates, to overcome instrumental error.

Chlorophyll content was measured with the portable chlorophyll content meter CL-01 (Hansatech Instruments Ltd., Pentney, King’s Lynn, Norfolk, UK). The device is equipped with two LED light sources at 620 nm and 940 nm; the relative chlorophyll content range varies between 0 and 2000 units. Calibration and temperature compensation are automatic. Chlorophyll content is expressed in a logarithmic scale through the SPAD index. The photochemical reflectance index (PRI) was measured using a Plant Pen PRI 200 (Photon System Instrument, Drásov, Czech Republic). The device has an internal dual wavelength light source and measures in two narrow wavelength bands, centered close to 531 nm and 570 nm with a +/−5 nm tolerance. The Normalized Difference Vegetative Index was measured with the Plant Pen NDVI 300 (Photon System Instrument, Drásov, Czech Republic), with an internal dual wavelength light source, one in the visible (VIS: 625 nm–645 nm) and one in the near-infrared range (NIR: 750 nm–760 nm), with a +/−5 nm tolerance. Individual surface leaf temperature (SLT) was measured using a non-contact infrared thermometer, SCANTEMP 440 (Dostmann Electronic, Wertheim-Reicholzheim, Germany).

### 4.3. Yield and Quality Trait Evaluation

Berry quality traits (soluble solids, acidity, and pH) were analyzed in terms of must, as well as berry weight. Berry collection was performed for all genotypes in three complete blocks, in the three seasons. A sample of 60 berries per plot (experimental unit) was collected the day before harvest. In the laboratory, the berries from each plot were counted, weighted, and grape must was obtained from them. The analyses of the must were performed by standard methods: soluble solids by refractometry (probable alcohol by conversion) and acidity by titration.

### 4.4. Data Analysis

Linear mixed models were fitted to analyze the data following the experimental design of the field trial, a resolvable row–column design [27]. The models included the genotype effects, the resolvable replicate effects (including the effect of day), the within-replicate column effects (including the effect of time of day), the within-replicate row effects, and the random errors associated with the observations. For yield data, the mean yield of the experimental unit (plot) was used for each year. For physiological traits (NDVI, PRI, SPAD, and SLT), individual measurements were taken on different leaves in the same plot, so that the effect of the plot was added in the fitted models. In all cases, model effects (except the overall mean) were assumed to be independent and identically distributed normal variables with zero mean and corresponding variances. All random effects were assumed to be independent of each other. Additionally, in consideration of the data availability from three consecutive years in a perennial crop, the fitted model for each trait was a multivariate mixed model [38]. This model assumed an unstructured covariance matrix between years. Consequently, the repeated nature of the data was taken into account, and the genetic correlation between years was also assessed. The latter correlation is a measure of the genotype x year interaction (see [39]).

The covariance parameters were estimated by the restricted maximum likelihood method [40] and were tested using residual maximum likelihood ratio tests. Mixed models were fitted with the package ASReml-R [41] for software R(Version 4.2) [42].

From this methodology, an analysis was carried out using the indicators described below.

The coefficient of genotypic variation (${CV}_{G}$, in percentage) for each trait and year was calculated as the ratio between the estimate of the genotypic standard deviation and the overall mean of the trait to allow for the comparison of intra-varietal variability between traits.

A generalized measure of heritability was obtained for each trait and year, based on the prediction error variance of genotypic effects and the estimate of the genotypic variance component [43] to understand what proportion of the total observed variability of the field trial is explained by genetic causes.

The percentage of each design effect variance (resolvable replicates, rows within resolvable replicates, and columns within resolvable replicates) in the total design effect variance was calculated to understand the importance of experimental design effects for the assessment of a given trait.

For each trait, the estimates of the genetic correlations between years were used to assess clone x year interaction: a high positive genetic correlation indicates that the clones respond almost equally to environmental year differences, whereas a low genetic correlation indicates that the genotypes show a higher interaction with the environment (year).

For all years, the empirical best linear unbiased predictors (EBLUPs) of the genotypic effects of all the traits studied were obtained through mixed model equations [44] to provide information about the genetic component affecting these traits. The EBLUPs of the genotypic effects and the predicted genotypic values (PGVs) were both ranked to evaluate the most abiotic-stress-tolerant clones, corresponding to the lowest values of SLT and the highest values of NDVI, PRI, and SPAD.

### 4.5. Polyclonal Selection

Groups of the ten most tolerant clones were selected based on abiotic stress indicators (polyclonal selection) and the predicted genetic gains for all other traits were obtained (mean of the EBLUPs of the genotypes of the selected group as a percentage of the mean of the trait in the field trial) to assess the abiotic stress indicator (s) that is (are) more efficient in measuring abiotic stress tolerance.

Based on the abiotic stress indicator (s) that proved to be more efficient in identifying abiotic stress tolerance, final groups of 10 clones were selected (polyclonal selection) and the predicted genetic gains were calculated for all other traits. To provide a more precise characterization of the final polyclonal group selected, the data utilized for the prediction of genetic gains for the traits were based on the average from all years of evaluation available in the field trial: stress indicators, 2019–2021; yield, 2019–2023; and berry traits, 2019, 2020, 2021, and 2023).

Approximate prediction intervals (at 95% confidence) for genetic gain were calculated as the predicted genetic gain plus or minus 1.96 times the approximate prediction standard error associated with the mean of the EBLUPs of the genotypic effects of the selected polyclonal group.

## 5. Conclusions

Several stress indices were measured in clones of the variety Arinto for three growing seasons and all of them were used individually to select genotypes. Both chlorophyll related indices, NDVI and SPAD, did not perform well in the more stressful seasons, and in the less stressful one, correlated with vigor. The index PRI, associated with the functioning of the xanthophyll cycle to scavenge ROS, was able to select stress-tolerant clones. Its use led to some gains in yield and no effect on berry quality. The parameter SLT, that gives an indication of stomatal functioning, identifies the clones that are able to keep stomata open during stress, thus correlating positively with yield. This parameter led to gains in yield and to no changes in berry quality. Both parameters were able to identify tolerance to stress, however, due to the very low range of values of PRI, the correlations between seasons were close to one, an effect of the lack of variability. Due to this constraint, SLT appears again to be the most robust and reliable parameter to assess tolerance to stress in large populations of clones and to perform selection for higher tolerance to heat and/or drought. As a next step in this ongoing research, it would be interesting to understand the molecular basis for this tolerance, to identify molecular markers of tolerance that could expedite the process of selection.

## Figures and Tables

**Table 1:** For each trait in the three years of analyses, the overall mean, the estimate of the genotypic variance component $\left({\hat{\sigma }}_{g}^{2}\right)$ with the respective *p*-value of the REML log-likelihood ratio test for genotypic variance, the coefficient of genotypic variation (CVG %), the broad-sense heritability (H2), and the percentage of each design effects variance (resolvable replicates—Rep, rows within replicate—Row, and columns within replicate—Col) in the total design effects variance, are shown.

Trait	Year	Overall Mean	${\hat{\sigma }}_{g}^{2}$ (*p*-Value)	*CV_G_* (%)	$H^{2}$	Percentage of Design Effects Variance		
						Rep	Row	Col
NDVI	2019	0.68	0.0001 (<0.001)	1.08	0.563	50.86	4.44	44.71
	2020	0.79	0.00002 (<0.022)	0.53	0.223	48.50	0	51.50
	2021	0.85	0.00004 (0.019)	0.78	0.241	22.52	1.25	76.23
PRI	2019	0.02	0.000006 (<0.001)	13.49	0.595	32.80	4.88	62.33
	2020	0.02	0.0000007 (<0.001)	4.26	0.361	44.21	0	55.79
	2021	0.13	0.00014 (<0.001)	9.09	0.310	30.19	0.55	69.26
SPAD	2019	14.28	1.7424 (<0.001)	9.25	0.672	45.63	7.52	46.85
	2020	16.95	2.5165 (<0.001)	9.36	0.667	74.86	3.65	21.49
	2021	10.83	0.8025 (<0.001)	8.28	0.520	7.89	1.06	91.04
SLT (°C)	2019	31.16	1.4885 (<0.001)	3.92	0.583	70.11	1.90	27.99
	2020	29.37	0.5949 (<0.001)	2.63	0.617	58.06	5.96	35.98
	2021	25.06	0.3384 (<0.001)	2.32	0.478	61.18	6.28	32.54
Yield (kg plant^−1^)	2019	4.55	1.0743 (<0.001)	22.78	0.695	46.27	13.96	39.78
	2020	5.69	1.0949 (<0.001)	18.39	0.636	79.88	3.54	16.58
	2021	5.55	3.1761 (<0.001)	26.56	0.744	50.04	7.11	42.85
Berry weight (g)	2019	1.09	0.0064 (<0.001)	7.31	0.482	70.10	4.90	24.98
	2020	1.55	0.0136 (<0.001)	7.54	0.612	11.86	11.59	76.55
	2021	1.50	0.0070 (<0.001)	5.57	0.457	9.43	31.80	58.77
Berry soluble solids (°Brix)	2019	22.90	1.1441 (<0.001)	4.67	0.668	82.74	5.78	11.49
	2020	23.07	1.2403 (<0.001)	4.83	0.692	5.05	7.5	87.44
	2021	23.51	0.2324 (<0.001)	2.05	0.478	52.77	24.58	22.64
Berry total acidity (tartaric acid, g L^−1^)	2019	5.60	0.3898 (<0.001)	11.15	0.714	45.51	10.92	43.57
	2020	4.39	0.1084 (<0.001)	7.49	0.668	38.06	0	61.94
	2021	6.93	0.1566 (<0.001)	5.71	0.493	84.37	0	15.63
Berry pH	2019	3.59	0.0065 (<0.001)	2.25	0.648	14.95	1.23	83.82
	2020	3.69	0.0039 (<0.001)	1.69	0.486	0	0	100
	2021	3.54	0.0026 (<0.001)	1.45	0.575	8.11	4.69	87.20

**Table 2:** Empirical best linear unbiased predictors (EBLUPs) of genotypic effects for all traits and years in the clones with the highest (maximum) and lowest (minimum) values and the predicted genotypic value (PGV) of the clones with the highest (maximum) and lowest (minimum) values, obtained with the fitting of the linear mixed models.

Trait	Year	EBLUP of Genotypic Effect		PGV	
		Minimum	Maximum	Minimum	Maximum
NDVI	2019	−0.030	0.011	0.648	0.689
	2020	−0.007	0.005	0.783	0.795
	2021	−0.009	0.019	0.845	0.873
PRI	2019	−0.013	0.003	0.006	0.022
	2020	−0.002	0.001	0.018	0.021
	2021	−0.035	0.013	0.095	0.141
SPAD	2019	−3.284	2.317	10.98	16.58
	2020	−4.164	2.416	12.78	19.36
	2021	−1.716	1.510	9.86	13.09
SLT (°C)	2019	−2.627	2.415	28.531	33.571
	2020	−1.662	1.507	27.746	30.915
	2021	−0.945	1.561	24.120	26.627
Yield (kg plant^−1^)	2019	−2.072	2.860	2.48	7.41
	2020	−2.909	2.373	2.78	8.06
	2021	−3.312	4.303	2.24	9.85
Berry weight (g)	2019	−0.124	0.230	0.968	1.322
	2020	−0.493	0.203	1.056	1.753
	2021	−0.190	0.147	1.310	1.647
Berry Soluble Solids (ºBrix)	2019	−2.285	1.701	20.61	24.60
	2020	−2.718	1.465	20.35	24.53
	2021	−1.013	1.037	22.50	24.55
Berry Total Acidity (tartaric acid, g L^−1^)	2019	−0.884	2.737	4.715	8.336
	2020	−0.463	1.542	3.930	5.936
	2021	−0.638	0.907	6.289	7.833
Berry pH	2019	−0.181	0.194	3.412	3.787
	2020	−0.103	0.107	3.588	3.798
	2021	−0.122	0.104	3.423	3.649

**Table 3:** Genetic correlation estimate (±SE) of the abiotic stress indicators (SLT, NDVI, SPAD, and PRI) and of yield and berry traits (soluble solids, pH, total acidity, and weight) between the three seasons, obtained with the fitting of the multivariate linear mixed models.

Stress Indicator/Trait	2019–2020	2019–2021	2020–2021
SLT (°C)	0.404 ± 0.113	0.213 ± 0.166	0.154 ± 0.158
NDVI	*	0.621 ± 0.210	0.792 ± 0.302
PRI	*	0.766 ± 0.137	*
SPAD	*	*	*
Yield (kg plant^−1^)	0.783 ± 0.067	0.904 ± 0.039	0.845 ± 0.058
Soluble solids (°Brix)	0.866 ± 0.074	0.734 ± 0.106	0.914 ± 0.100
Berry total acidity (tartaric acid, g L^−1^)	0.836 ± 0.062	0.756 ± 0.099	0.794 ± 0.089
pH	0.797 ± 0.114	0.672 ± 0.098	0.839 ± 0.117
Berry weight	0.281 ± 0.142	0.796 ± 0.148	0.628 ± 0.118

*: No value due to convergence problems revealed by the multivariate mixed model fitted to estimate the correlations.

**Table 4:** Predicted genetic gains of the selected group of ten genotypes (polyclonal selection) for each abiotic stress indicator and for yield and quality characteristics when selection is performed according to each of those indicators in the three seasons studied.

Traits		Predicted Genetic Gain (as Percentage of the Mean of the Variety)		
		2019	2020	2021
NVDI	NVDI	1.165 *	0.468 *	0.747 *
	Yield (kg plant^−1^)	3.761	−2.265	13.756 *
	Berry weight (g)	2.665	1.641	−0.127
	Berry pH	1.400 *	0.727	−0.245
	Berry soluble solids (°Brix)	2.833 *	2.066 *	0.327
	Berry total acidity (tartaric acid, g L^−1^)	−3.506	−1.985	−1.590
PRI	PRI	14.719 *	4.090 *	8.212 *
	Yield (kg plant^−1^)	7.429	−2.190	2.624
	Berry weight (g)	−2.062	0.094	1.475
	Berry pH	−0.846 *	0.013	−0.209
	Berry soluble solids (°Brix)	−1.521	2.108 *	−0.039
	Berry total acidity (tartaric acid, g L^−1^)	0.100	−1.556	0.259
SPAD	SPAD	13.280 *	11.358 *	11.220 *
	Yield (kg plant^−1^)	−2.022	−0.622	4.611
	Berry weight (g)	2.120	1.257	−0.516
	Berry pH	1.012 *	0.720	0.015
	Berry soluble solids (°Brix)	0.636	3.223 *	0.223
	Berry total acidity (tartaric acid, g L^−1^)	−2.218	−2.472	−1.003
SLT	SLT (°C)	−6.142 *	−4.316 *	−3.107 *
	Yield (kg plant^−1^)	14.774 *	6.513	−3.314
	Berry weight (g)	1.044	0.818	−0.675
	Berry pH	0.919 *	−0.094	0.035
	Berry soluble solids (°Brix)	2.462 *	0.932	0.038
	Berry total acidity (tartaric acid, g L^−1^)	−5.077 *	−0.583	−0.004

*: If the range of values for the approximate prediction interval (at 95% confidence) does not include zero.

**Table 5:** Predicted genetic gains (as a percentage of the mean of the variety in the field trial) of the polyclonal selection for SLT from the analysis using the mean values from the three seasons. The global averages of the predicted genetic gains for all abiotic stress indicators, yield, and berry characteristics are shown, as well as the corresponding approximate prediction intervals (at 95% of confidence).

Trait	Average Predicted Gain	Prediction Intervals
SLT (°C)	−3.131	[−3.892, −2.371]
SPAD	3.239	[0.504, 5.974]
NDVI	0.139	[−0.141, 0.420]
PRI	0.035	[−3.078, 3.148]
Yield (kg plant^−1^)	7.319	[1.499, 13.140]
Berry weight (g)	−1.295	[−3.346, 0.755]
Berry pH	0.374	[−0.122, 0.870]
Berry Soluble Solids (°Brix)	1.373	[0.385, 2.361]
Berry Total Acidity (tartaric acid g L^−1^)	−1.070	[−3.220, 1.080]

## Data Availability

The original contributions presented in the study are included in the article/supplementary material; further inquiries can be directed to the corresponding author/s.

## Supplementary Materials

The following supporting information can be downloaded at: https://www.mdpi.com/article/10.3390/plants14162480/s1, Figure S1: Monthly maximum (T max) and minimum (T min) temperatures and total monthly precipitation (Rainfall) in the seasons of 2019 (a), 2020 (b), and 2021 (c).; Figure S2: Pearson correlation coefficient between the predicted genotypic values (PGVs) of all the traits analyzed in 2019, 2020, and 2021. The significance of the correlations is indicated by the color code, as shown on the legend.; Table S1: Empirical Best Linear Unbiased Predictors (EBLUPs) of Genotypic Effects for Surface Leaf Temperature (SLT, °C), SPAD, PRI, and NDVI, Yield (Kg plant^−1^), and berry traits (°Brix, pH, total acidity, and weight) for the three years (2014, 2015, 2016) (obtained with the fitting of the multivariate linear mixed model). Table S2: Predicted Genotypic Values (PGVs) for Surface Leaf Temperature (SLT, °C), SPAD, PRI, and NDVI, Yield (Kg plant^−1^), and berry traits (°Brix, pH, total acidity, and weight) and respective rank for tolerance to abiotic stress, for the three seasons (2019, 2020, and 2021) (obtained with the fitting of the multivariate linear mixed model). In the columns of SLT, SPAD, PRI, and NDVI, the clones highlighted in green are the 10 most tolerant clones and the ones highlighted in yellow are the 10 most sensitive ones, selected using the respective abiotic stress indicator, and as represented in Figure 1. Table S3: Results obtained for the cultural traits, based on the average of the years where the broad-sense heritability (H^2^) was greater than 0.40. For each trait, the table shows the overall mean, the estimate of the genotypic variance component, with the respective *p*-value of the REML log likelihood ratio test for genotypic variance, the coefficient of genotypic variation (CV_G_, in %), and the value of broad-sense heritability (H^2^). These parameters were calculated for the average of three years (2019 to 2021) for abiotic stress indicators, of four years (2019, 2020, 2021, and 2023) for berry traits, and of five years (2019 to 2023) for yield.
