# Supplementary material for: Intra-Varietal Variability for Abiotic Stress Tolerance Traits in the Grapevine Variety Arinto
Source: Plants (Basel). 2025 Aug 10;14(16):2480. doi: 10.3390/plants14162480 (PMC12389634; doi:10.3390/plants14162480)
Supplement: Supplementary file 1 [file plants-14-02480-s001.zip › SuplementalFigures.pdf]

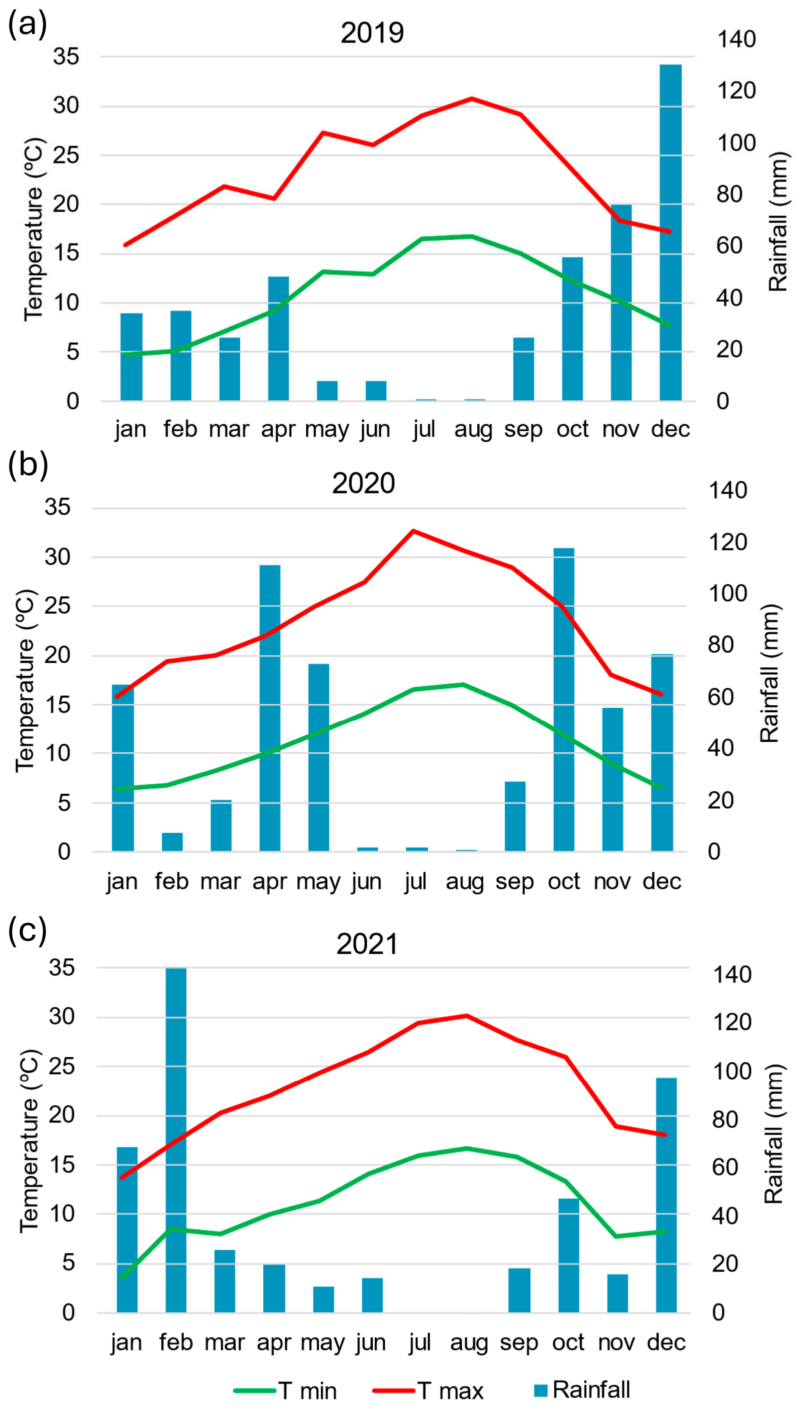

Figure S2 | Monthly maximum (T max) and minimum (T min) temperatures and total monthly precipitation (Rainfall) in the seasons of 2019 (a), 2020 (b), and 2021 (c).

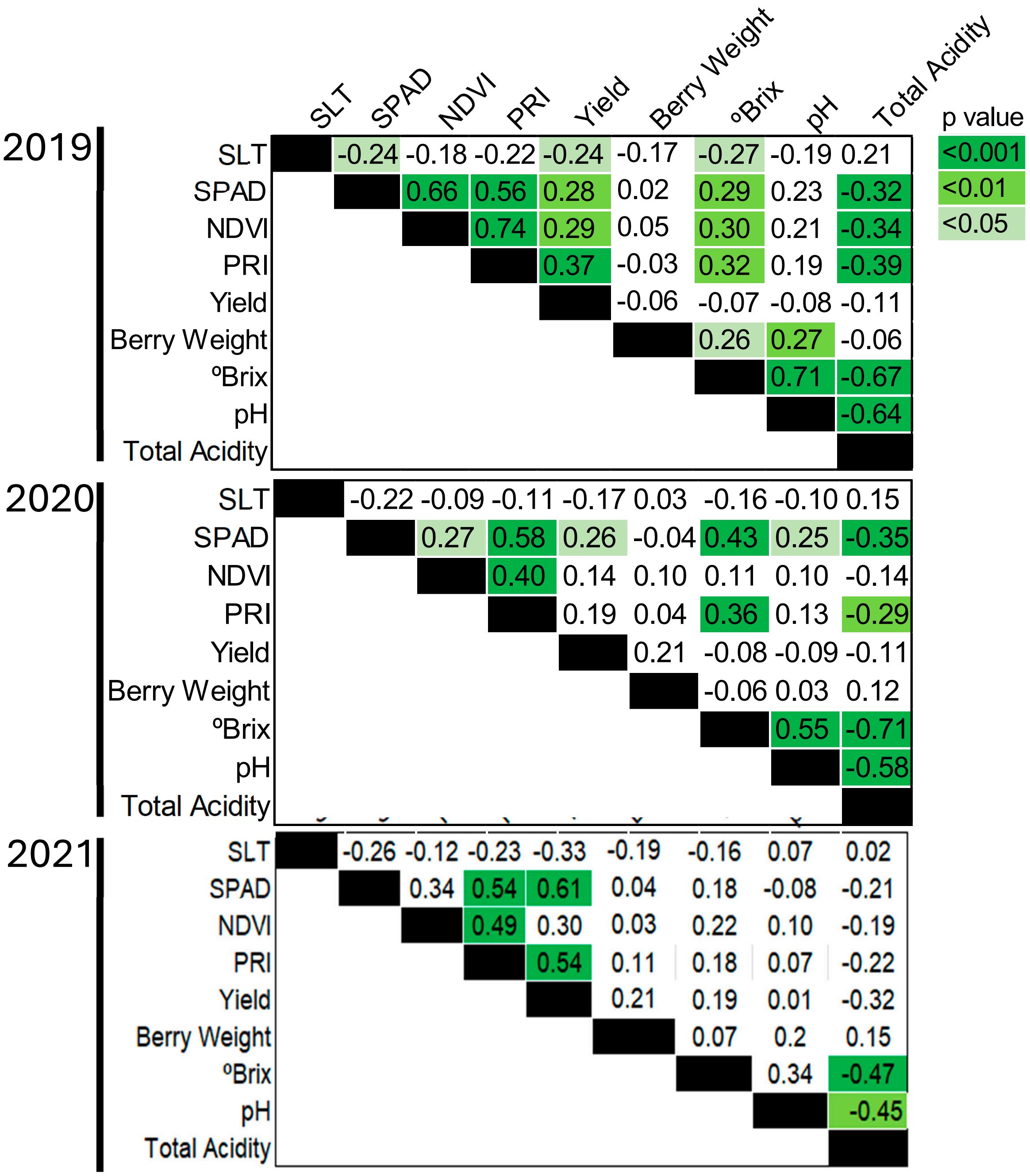

Figure S1 | Pearson correlation coefficient between the predicted genotypic values (PGVs) of all the traits analyzed in 2019, 2020, and 2021. Significance of the correlations is indicated by the color code, as shown on the legend.
